# Changes in the Resting-State Cortical Oscillatory Activity 6 Months After Modified Tinnitus Retraining Therapy

**DOI:** 10.3389/fnins.2019.01123

**Published:** 2019-10-18

**Authors:** Sang-Yeon Lee, Jihye Rhee, Ye Ji Shim, Yoonjoong Kim, Ja-Won Koo, Dirk De Ridder, Sven Vanneste, Jae-Jin Song

**Affiliations:** ^1^Department of Otolaryngology-Head and Neck Surgery, Seoul National University Bundang Hospital, Seongnam, South Korea; ^2^Department of Otolaryngology-Head and Neck Surgery, Seoul Veterans Hospital, Seoul, South Korea; ^3^Department of Otolaryngology-Head & Neck Surgery, Seoul National University Hospital, Healthcare System Gangnam Center, Seoul, South Korea; ^4^Unit of Neurosurgery, Department of Surgical Sciences, Dunedin School of Medicine, University of Otago, Dunedin, New Zealand; ^5^Laboratory for Clinical and Integrative Neuroscience, School of Behavioral and Brain Sciences, The University of Texas at Dallas, Dallas, TX, United States

**Keywords:** tinnitus retraining therapy, quantitative electroencephalography, neurophysiological model, cortical power, connectivity

## Abstract

Although tinnitus retraining therapy (TRT) based on Jastreboff’s classical neurophysiological model is efficacious in most patients, its effects on the cortical activity changes responsible for the improvement of tinnitus are still unclear. In this study, we compared pre- and post-TRT resting-state quantitative electroencephalography (rs-qEEG) findings to identify power changes that could explain TRT-induced improvements. Thirty-seven patients with severe tinnitus were enrolled in the study, and rs-qEEG data recorded before the initial TRT sessions and 6 months after TRT were compared. In addition, associations between the changes in qEEG and percentage improvements in Tinnitus Handicap Inventory (THI) scores and numeric rating scale (NRS) scores of tinnitus loudness and tinnitus perception were examined. The mean THI score decreased significantly 6 months after the initial TRT session. Also, significant improvements were observed 6 months after the initial TRT session compared with the pre-treatment scores in NRS loudness, distress, and perception. As compared with the pre-TRT status, post-TRT 6 months status showed significantly decreased powers in the left primary and secondary auditory cortices for the gamma frequency band. Changes in the alpha 1 frequency band power in the right insula and orbitofrontal cortex (OFC) appeared to be positively correlated with the percentage changes in NRS distress. These results suggested that TRT improved tinnitus-related distress by reducing the power of the top-down autonomic response modulator or peripheral physiological responses to emotional experiences. That is, TRT induced habituation via modulation of functional connections between the auditory system and the limbic and autonomic nervous systems. Our results confer additional basis for understanding the neurophysiological model and the newly suggested integrative model of tinnitus by De Ridder et al. (2014) in the context of the long-term efficacy of TRT.

## Introduction

Subjective tinnitus is a common otological symptom characterized by the conscious auditory perception in the absence of an external stimulus, which is often called a “phantom sound” as there is no corresponding genuine physical source of the sound (Jastreboff, 1990). Although the precise mechanism remains unclear, the current consensus is that tinnitus may be a result of central maladaptive plastic changes reflecting a complex interplay of auditory and non-auditory brain regions responsible for tinnitus generation and tinnitus-related distress. That is, tinnitus is associated with functional changes not only in the auditory cortex but also in non-auditory regions such as the limbic, frontal, and parietal areas (De Ridder et al., 2011a; Adjamian et al., 2014; Lee et al., 2017).

Several management options, such as tinnitus retraining therapy (TRT), cognitive behavioral therapy, sound therapy, hearing rehabilitation using hearing aids or cochlear implants, pharmacotherapy, and brain stimulation, have been suggested for tinnitus (Langguth et al., 2013; Song et al., 2017). Of these modalities, TRT is a combination of directive counseling and sound therapy that seeks to manage tinnitus and reduce sound tolerance (Jastreboff, 1990). TRT is based on Jastreboff’s classical neurophysiological model suggesting that the detection of tinnitus is made by neural network mechanisms, whereas the perception and evaluation involve the auditory cortical areas, limbic system, and sympathetic part of the autonomic nervous system (Jastreboff, 1999, 2007, 2015; Jastreboff and Jastreboff, 2000, 2003, 2006). TRT aims to suppress tinnitus by extinguishing functional connections between the auditory, limbic, and autonomic nervous systems, thus creating habituation to tinnitus-evoked reactions and ultimately the phantom perception itself (Jastreboff, 2015).

More than 100 studies have explored the effectiveness of TRT, and the majority of these studies indicated that TRT improved tinnitus significantly in approximately 80% of patients (Jastreboff, 2015). This implication has also been supported by the Cochrane review suggesting TRT as an effective treatment for patients with tinnitus, despite the conclusion based upon a single, low-quality randomized controlled trial (Phillips and McFerran, 2010). Although most studies have reported the effectiveness of TRT, changes in the cortical power responsible for the improvement of tinnitus are still unclear. A recent study demonstrated the neural substrates predicting short-term improvement of tinnitus loudness and distress by correlating resting-state source-localized quantitative electroencephalography (rs-qEEG) findings and the extent of tinnitus improvement 3 months after the first TRT session (Kim et al., 2016). In this study, pre-TRT activities in several cortical areas related to tinnitus generation, parasympathetic activity, and the descending noise-canceling system have been suggested as possible predictors of short-term improvement of tinnitus after TRT (Kim et al., 2016). However, the possible role of TRT in modulating abnormal cortical activities and thereby improving tinnitus remains unclear because there have been no studies comparing pre- and post-TRT cortical activities and correlating the cortical power changes with the degree of symptom improvement.

As a follow-up to our previous work (Kim et al., 2016), we compared rs-qEEGs measured before TRT and 6 months after the first TRT session in the present study. In addition, we correlated the extent of tinnitus improvement with the changes in rs-qEEG signals to determine the cortical activity changes responsible for the improvement of tinnitus using source localization complimented by connectivity analysis.

## Materials and Methods

### Participants

The medical records of patients with subjective tinnitus who were enrolled in a modified TRT program at Seoul National University Bundang Hospital from May 2015 to April 2017 were retrospectively reviewed. Subjects with normal (<25 dB hearing level) or mild hearing loss (25–40 dB hearing level) calculated by averaging the pure tone thresholds at 0.5, 1, 2, and 3 kHz (Bae et al., 2017, 2018, 2019; Sunwoo et al., 2017; Song et al., 2018; Lee et al., 2019; Shim et al., 2019) on both ears were included in the present study. Subjects were excluded if they had the following conditions: (1) pulsatile tinnitus, Ménière’s disease, otosclerosis, any psychiatric or neurological disorders, histories of drug or alcohol abuse, and chronic headache; (2) a history of head injury that caused loss of consciousness; (3) a history of seizures; and (4) subjects who were lost to follow-up were excluded from the study. Accordingly, the final study population consisted of 37 patients with subjective tinnitus. The study was approved by the Institutional Review Board of the Clinical Research Institute at Seoul National University Bundang Hospital, and was conducted in accordance with the Declaration of Helsinki (IRB-B-1708-412-128). The requirement for patient consent was waived. At the initial visit, we assessed the characteristics of tinnitus in a structured manner including the affected side, the nature of symptoms (pure-tone or narrow-band noise), and symptom duration. All participants underwent pure-tone audiometry, psychoacoustic tinnitus tests, such as tinnitus pitch matching, tinnitus loudness matching, and the minimum masking level test (Henry and Meikle, 2000), and an initial pre-TRT rs-qEEG. The perceived tinnitus handicap was measured by the Tinnitus Handicap Inventory (THI) (Newman et al., 1996). The THI consists of a total of 25 items, consisting of a functional subscale (11 items), an emotional subscale (9 items), and a catastrophic subscale (5 items). The total THI score (0–100 points) is used to determine a measure of the overall severity of the tinnitus annoyance or general tinnitus-related distress (Newman et al., 1996). The numeric rating scale (NRS) scores for tinnitus loudness (answering the question “how loud is your tinnitus?” on a scale from 0 to 10), tinnitus-related distress (answering the question “how bothered are you by your tinnitus?” on a scale from 0 to 10), and the percentage of the daytime during which the participant perceived tinnitus (NRS tinnitus perception, 0 to 100%) were used to evaluate the difficulties associated with tinnitus. All tests were conducted before and 6 months after the initial TRT session.

### Modified TRT Program

After the initial evaluation, all subjects enrolled in the present study participated in our modified TRT program, which was based on the neurophysiological model of Jastreboff (Jastreboff, 1990, 1999, 2007, 2015; Jastreboff and Jastreboff, 2000, 2003, 2006). “The original TRT includes not only directive counseling and sound therapy but also stress management and facultative psychotherapeutic treatment. Meanwhile, the main point of modified TRT is the counseling, which based on the neurophysiological model of tinnitus (Mazurek et al., 2006).” During the initial session for 40 min, the subjects were educated regarding the background of tinnitus and TRT, such as the definition and incidence of tinnitus, simplified anatomy of the ear and auditory pathways, the subconscious processing and conscious perception of auditory stimuli, selective listening and the central noise-canceling mechanism (Song et al., 2015a), the neurophysiological model suggested by Jastreboff, tinnitus demystification, motivation and reassurance, explanation of habituation and ignoring tinnitus as the goal of TRT, the roles played by hearing aids and various sound therapies in treating tinnitus, and directed education on how to avoid silence and to add non-noxious sounds to the daily environment (i.e., ambient sound stimulation). All subjects were recommended to be exposed to ambient sound stimulation as much as possible and to avoid complete silence while they are awake. Hearing aids were not used in the present study population as only subjects with normal hearing or mild hearing loss were included to avoid bias with the cortical changes due to hearing loss and its improvement after hearing aid use. After the initial TRT session, all subjects were scheduled for monthly follow-up TRT sessions (30 min each). At each follow-up session, all subjects were reevaluated by follow-up THI and NRS score survey and counseled to determine subjective changes in their symptoms and to review the core content of the initial counseling session. In this way, a total of 7 TRT sessions were given to each subject.

### EEG Recording

Rs-EEGs were obtained before and 6 months after the initial TRT session in all 37 subjects. Before measurement, the participants were asked not to drink alcohol for 24 h and to avoid caffeinated beverages on the day of recording to minimize alcohol-induced changes in EEG (Korucuoglu et al., 2016) or caffeine-induced alpha and beta power decreases (Siepmann and Kirch, 2002). EEGs were recorded with the patient in the sitting position with the eyes closed for 5 min using a tin-electrode cap (Electro-Cap, Eaton, OH, United States), a Mitsar amplifier (EEG-201; Mitsar, St. Petersburg, Russia), and WinEEG software version 2.84.44 (Mitsar) in a fully lit room shielded from sound and stray electric fields. The EEG was sampled with 19 electrodes in the standard 10–20 International placement, referenced to linked ears, and impedances were maintained below 5 kΩ at each electrode throughout the EEG measurements. We imported EEG as epoched data, and the data were collected for 100 2-second epochs eyes closed. The epochs remained after artifact removal differed from person to person according to the amount of removed data. The sampling rate was 1024 Hz, and the high- and low-pass frequencies used were 0.15 and 200 Hz, respectively. Initially recorded data were then resampled to 128 Hz and band-pass filtered using a fast Fourier transform filter and application of a Hanning window at 2–44 Hz. After importing the data into Eureka! Software (Sherlin and Congedo, 2005), plotting of the EEG stream and manual inspection for artifacts, such as eye movement, unconscious blinking, teeth clenching, and any form of body movements that can affect EEG stream, were performed to remove all episodic artifacts. As the vigilance level of participants may affect test results, we carefully monitored abnormal EEG patterns, such as slowing of the alpha rhythm or the emergence of sleep spindles (Moazami-Goudarzi et al., 2010). No participants included in the present study exhibited such abnormal EEG patterns throughout the measurements.

### Source Localization Analysis

Low-resolution brain electromagnetic tomography (LORETA)-KEY software^1^ designed for functional localization of standardized current densities based on certain electrophysiological and neuroanatomical constraints (Pascual-Marqui, 2002) was used to localize the cortical sources that generated the activities recorded from the scalp electrode in each of the following 8 frequency bands: delta (2–3.5 Hz), theta (4–7.5 Hz), alpha 1 (8–10 Hz), alpha 2 (10–12 Hz), beta 1 (13–18 Hz), beta 2 (18.5–21 Hz), beta 3 (21.5–30 Hz), and gamma (30.5–44 Hz) (Kim et al., 2015; Song et al., 2015a, b, 2017; Han et al., 2018; Vanneste et al., 2018).

Estimation of the position of the current sources from electrode potentials is known as the inverse problem or the neural source imaging problem (Grech et al., 2008; Lopez Rincon and Shimoda, 2016). In other words, the inverse problem involves the source reconstruction of images from electric neuronal activities based on extracranial measurements. Among various techniques available for EEG source localization, LORETA algorithm gives the best solution in terms of both localization error and ghost sources (Grech et al., 2008). The software makes assumptions with regard to orientations and strengths of the neighboring neuronal sources that are represented by adjacent voxels. This software implements revisited realistic electrode coordinates (Jurcak et al., 2007) and the lead field produced by Fuchs et al. (2002) resulting from standard electrode positions readjusted to a standard Montreal Neurological Institute (MNI)-152 head in combination with a boundary element method derived from the same standard anatomy (Jurcak et al., 2007). The LORETA-KEY anatomical template divides and labels the neocortical MNI-152 volume, including the hippocampus and anterior cingulate cortex, into 6239 voxels with dimensions of 5 × 5 × 5 mm, based on the Daemon Atlas (Lancaster et al., 2000). Anatomical labeling of significant clusters was performed automatically using a toolbox implemented in LORETA-KEY. The locations of significant clusters were initially investigated using the Anatomy toolbox (Eickhoff et al., 2005), and reconfirmed using the Talairach and Tournoux atlas (Talairach and Tournoux, 1988).

### Functional Connectivity Analysis

Phase synchronization over multiple frequency bands has been proposed to be the most plausible mechanism of large scale neuronal integration overcoming the distributed anatomical and functional organization of brain activity to enable coherent behavior and cognition (Varela et al., 2001). Therefore, phase synchronization and the amount of coherence between pre- and post-TRT qEEGs in different regions of interest (ROIs) were calculated to analyze functional connectivity by the built-in LORETA-KEY connectivity toolbox. The toolbox determines coherence and phase synchronization between multivariate time series; the measures are expressed as the sums of lagged/instantaneous dependencies. Of various methods, we analyzed lagged coherence as it excludes non-lagged parts of coherence that comprises effects of volume conduction (artificially increasing coherence), and effects of non-recorded sources that simultaneously drive recorded sources (Milz et al., 2014).

A total of 28 ROIs defined by Brodmann area were selected as possible nodes based on the previous literature on tinnitus: the bilateral primary and secondary auditory cortices (Rolls, 2004; Kringelbach, 2005; Smits et al., 2007), the bilateral parahippocampal (Landgrebe et al., 2009), the bilateral rostral/pregenual/subgenual anterior cingulate cortices (r/pg/sgACCs) (Vanneste et al., 2010; De Ridder et al., 2011b; Golm et al., 2013), the bilateral ventral medial prefrontal cortices (vmPFC) (Leaver et al., 2011, 2012), the bilateral insulae, bilateral precunei, and bilateral orbitofrontal cortices (OFC) (Vanneste et al., 2010; De Ridder et al., 2011b; Golm et al., 2016).

### Statistical Analysis

Statistical non-parametric mapping (SnPM) was adapted for permutation tests for source localization and functional connectivity. SnPM yields results similar to those obtained with the statistical parametric mapping approach using a general linear model (GLM) with multiple comparison corrections derived from random field theory (Nichols and Holmes, 2002). That is, SnPM corrects for multiple tests performed for all voxels and all frequency bands. Due to its non-parametric nature, the validity of SnPM does not rely on any assumption of Gaussianity. In this regard, SnPM can be used to verify the validity of less computationally expensive parametric approaches and is suitable for functional neuroimaging studies involving multi-subjects or group analyses in small numbers of subjects (Nichols and Holmes, 2002). SnPM’s permutation for correction for multiple comparisons (5,000 permutations in the current study) has previously been proven similar to those obtained using a standard GLM approach with multiple comparisons corrections (Holmes et al., 1996; Nichols and Holmes, 2002).

In this way, we compared pre- and post-TRT rs-qEEGs and correlated the extent of tinnitus improvement with the changes in rs-qEEG signals with regard to source-localized activity and functional connectivity. In addition, the paired *t*-test was used to evaluate the changes in perceived THI and NRS scores 6 months after TRT using SPSS (version 20.0; SPSS Inc., Chicago, IL, United States). In all analyses, *P* < 0.05 was taken to indicate statistical significance. The *t*-tests were based on one-tailed hypotheses.

## Results

### Demographic and Clinical Characteristics of the Subjects

The clinical characteristics of the 37 subjects enrolled in this study are summarized in Table 1. The mean THI score of the subjects was 56.3 ± 16.8, and the enrolled subjects’ tinnitus handicap ranged from “moderate” to “severe” as defined by Newman et al. (1998). The mean age of the subjects was 55.0 ± 11.9 years (range, 22–76 years), and 20 of the 37 subjects were male. Of the 37 subjects, 21 complained of unilateral tinnitus (15 with right tinnitus and 6 with left tinnitus), while the remaining 16 had bilateral tinnitus. Twenty-seven patients presented pure-tone tinnitus, while the other 10 presented narrow-band noise tinnitus. The mean symptom duration was 5.0 ± 4.1 years (range, 1–25 years).

**TABLE 1:** Demographic and Clinical characteristics.

**Parameters**	**Enrolled subjects (*n* = 37)**
**Sex**	
Male	20 (54.1%)
Female	17 (45.9%)
**Age (year)**	
Mean [SD]	55.0 [11.9]
Range	22–76
**Tinnitus laterality**	
Unilateral	21 (56.8%)
Bilateral	16 (43.2%)
**Tinnitus characteristics**	
Pure tone	27 (73.0%)
Narrow band noise	10 (27.0%)
**Duration (year)**	
Mean	5.0 [4.1]
Range	1–25
**Hearing level (dB)**	
Normal	26 (70.2%)
Mild hearing loss	11 (29.8%)

### Improvement in Questionnaire Scores 6 Months After the Initial TRT Session

As summarized in Table 2, the mean THI score improved significantly from 56.3 ± 16.8 to 37.5 ± 24.3 (*t* = 5.83, *P* < 0.001, Cohen’s *d* = 1.118, paired *t* test) 6 months after the initial TRT session. In addition, significant improvements were observed 6 months after the initial TRT session compared with the pre-treatment scores in NRS loudness (from 6.9 ± 1.8 to 6.0 ± 2.0, *t* = 3.525, Cohen’s *d* = 0.484, *P* = 0.007, paired *t* test), distress (from 7.0 ± 2.0 to 5.1 ± 2.6, *t* = 3.096, Cohen’s *d* = 0.916, *P* < 0.001, paired *t* test), and perception (from 85.9 ± 22.2% to 64.2 ± 29.4%, *t* = 3.451, Cohen’s *d* = 0.981, *P* < 0.001, paired *t* test).

**TABLE 2:** Changes in the perceived tinnitus handicap scores 6 months after TRT.

**Parameters**	**Pre-TRT**	**Post-TRT 6 months**	***P*-value**	**Cohen’s *d***
THI (0–100)	56.3 [SD:16.8]	37.5 [SD:24.3]	<0.001	1.118
NRS perception (%)	85.9 [SD:22.2]	64.2 [SD:29.4]	0.007	0.484
NRS loudness (0–10)	6.9 [SD:1.8]	6.0 [SD:2.0]	<0.001	0.916
NRS distress (0–10)	7.0 [SD:2.0]	5.1 [SD:2.6]	<0.001	0.981

*TRT, tinnitus retraining therapy; NRS, numeric rating scale.*

### Source-Localized Cortical Power and Connectivity Comparison Between Pre- and Post-TRT Conditions

**FIGURE 1:**
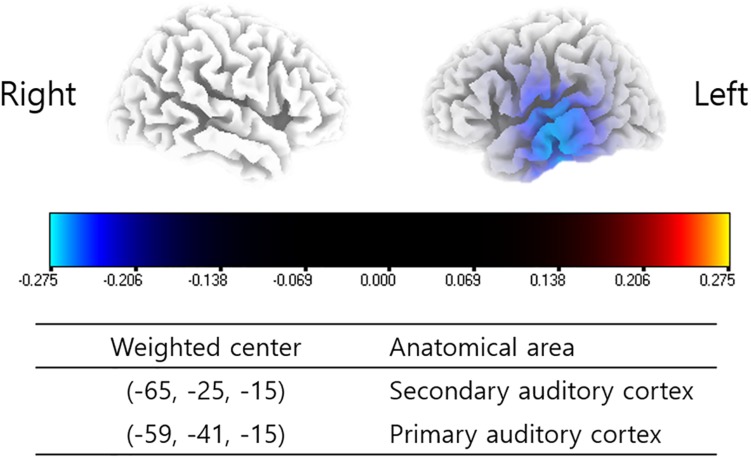
Source-localized cortical power comparison between pre- and post-TRT conditions using repeatedly measured quantitative electroencephalography (qEEG) data. Post-TRT qEEG data showed decreased powers in the left primary and secondary auditory cortices (A1 and A2) for the gamma frequency band as compared with pre-TRT data.

The subjects enrolled in the present study showed significantly decreased powers in the left primary and secondary auditory cortices (A1 and A2) for the gamma frequency band 6 months after TRT in comparison with pre-TRT status (*P* < 0.01) (Figure 1). There were no other statistically significant differences between the pre- and post-TRT rs-qEEGs for the delta, theta, alpha 1 and 2, and beta 1–3 frequency bands.

No significant differences were found between pre- and post-TRT rs-qEEG data with regard to connectivity among the 28 ROIs.

### Correlation Analyses Between NRS Changes and Source-Localized Cortical Power Changes

**FIGURE 2:**
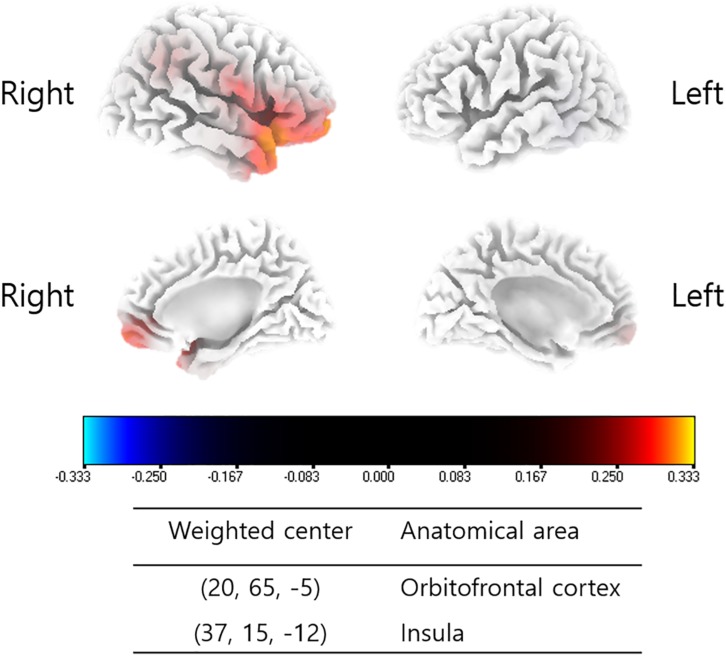
Source-localized correlation analysis between the percentage improvements in NRS of tinnitus distress and changes in TRT (pre- and post-TRT) resting-state qEEG data. EEG power changes in the right insula and orbitofrontal cortex (OFC) for the alpha 1 frequency band showed a positive correlation with the percentage changes in NRS distress (*P* = 0.04).

Changes in the source-localized cortical power in the right insula and OFC for the alpha 1 frequency band were positively correlated with the percentage improvements in NRS distress (*P* = 0.04) (Figure 2). Meanwhile, percentage improvements in NRS loudness or NRS perception showed no significant correlations with changes in source-localized cortical power after TRT.

### Correlation Analyses Between Changes in NRS Parameters and Connectivity Changes

No significant correlations were observed in the percentage improvements in NRS loudness/distress/perception and connectivity among the 28 ROIs.

## Discussion

This study was performed to explore the role of modified TRT with regard to the modulation of abnormal cortical activities in subjects with debilitating tinnitus 6 months after the initial session by analyzing changes in resting-state cortical activities. By analyzing repetitively measured rs-qEEGs, we demonstrated that the cortical power of tinnitus subject changes after repeated modified TRT sessions and the improvement in symptoms is correlated with the changes in resting-state cortical oscillatory activities. To our knowledge, although the classical neurophysiological model of tinnitus suggested by Jastreboff postulated the involvement of cortical structures, such as the auditory cortex and limbic system, this is the first study to elucidate the cortical changes that may be relevant to the improvement of tinnitus after successful TRT sessions in subjects with severe tinnitus.

### Sustained Improvement in Questionnaire Scores 6 Months After the Initial TRT Session

As a follow-up to our previous report (Kim et al., 2016), the present study was performed in a population of 37 patients enrolled in the TRT program of our institute and followed up for 6 months after the initial TRT session. As summarized in Table 2, the improvements in mean THI score (from 56.3 ± 16.8 to 37.5 ± 24.3), NRS loudness (from 6.9 ± 1.8 to 6.0 ± 2.0), distress (from 7.0 ± 2.0 to 5.1 ± 2.6), and perception (from 85.9 ± 22.2% to 64.2 ± 29.4%) indicated significant sustained improvements 6 months after the initial TRT session compared with the pre-treatment scores. The results were comparable to our previous preliminary report (Kim et al., 2016) as well as to other previous studies (Jastreboff and Jastreboff, 2006; Jastreboff, 2015).

### Abnormal Auditory Cortical Power Is Suppressed 6 Months After Initial TRT Session

The perception of tinnitus has previously been suggested to be linked to increased neural activity in the auditory cortex, and A1 and A2 has been suggested to play important roles in tinnitus perception (De Ridder et al., 2015), and these areas have recently been suggested as a part of tinnitus core; i.e., the minimal brain area required to generate the phantom sound (De Ridder et al., 2014), along with the parahippocampus (PHC). Indeed, a recent meta-analysis of PET studies reported increased regional cerebral blood flow in A1 and A2 in tinnitus patients in comparison with normal controls, which supports the suggestion that A1 and A2 are important areas for tinnitus perception (Song et al., 2012). However, the included studies in this meta-analysis did not control for hearing loss, thereby might have been demonstrating correlates of hearing loss as well as, or instead of, tinnitus. Therefore, this awaits further confirmation.

Tinnitus was suggested to be caused by abnormal, spontaneous, and constant gamma-band activity generated as a consequence of hyperpolarization of the thalamic nuclei (Llinás et al., 1999). On the other hand, qEEG (Van Der Loo et al., 2009; Vanneste and De Ridder, 2012; Meyer et al., 2014) and MEG studies (Weisz et al., 2005, 2007) showed that tinnitus perception and its loudness are correlated with sustained high-frequency gamma-band activity in the auditory cortices. These results suggest that increased gamma-band activity in the auditory cortices is associated with the perception and loudness of tinnitus. The present study demonstrated significantly decreased gamma power at the left A1 and A2 at post-TRT 6 months as compared with pre-TRT baseline, showing decreased cortical power of the core tinnitus generator, i.e., the auditory cortices, after TRT. However, of note, reports on auditory cortical gamma power changes in patients with tinnitus appear to be controversial. For instance, other previous qEEG or MEG studies have failed to find gamma power changes between tinnitus- and control groups (Adjamian et al., 2012; Song et al., 2015b). Moreover, Sedley et al. revealed the differential patterns of gamma-band oscillations in the auditory cortex according to residual inhibition or excitation, suggesting a mutual inhibitory role of gamma oscillations on tinnitus symptoms (Sedley et al., 2012). These discrepant findings might be attributable to highly variable gamma-band frequency across studies (Weisz et al., 2007; Van Der Loo et al., 2009; Song et al., 2015b) and confounders such as laterality and hearing loss (Ortmann et al., 2011), and therefore further studies to elucidate the role of gamma power changes in the auditory cortex should be performed. Furthermore, reduced gamma power in the left A1 and A2 6 months after the initial session of modified TRT might also be biased by an effect of time, familiarity with EEG, or an indirect correlate of these such as attention. Future studies in a larger number of subjects should be performed to reconfirm our current findings.

Our preliminary study indicated that the percentage changes in improvement in tinnitus loudness were correlated with the gamma-band activities of A1 and A2 3 months after TRT (Kim et al., 2016). In this regard, decreased activities in the A1 and A2 in patients with tinnitus after TRT may be related to both short- and long-term TRT-mediated improvements in tinnitus. In addition, in a recent rs-qEEG study performed by Vanneste and De Ridder (2016), tinnitus was suggested to be more closely related to auditory cortical activity in patients with little or no hearing loss, but not to parahippocampal activity, whereas tinnitus appears to be related to parahippocampal mechanisms in those with more severe hearing loss. As the subjects enrolled in the present study had no or minimal hearing loss with thresholds better than 40 dB bilaterally, the deactivation of the auditory cortices in our subjects after TRT were consistent with that reported by Vanneste and De Ridder (2016).

### TRT Attenuated Tinnitus-Related Distress via Modulation of the Interaction of the Limbic and Autonomic Nervous Systems

According to the classical neurophysiological model of tinnitus (Jastreboff, 1990, 2015), once an abnormal activity that causes tinnitus is classified as important, it spreads to the limbic system and autonomic nervous system. That is, tinnitus-related distress is attributable to changes in the limbic system and the autonomic nervous system as well as the interaction between these two systems. In an integrative model of tinnitus suggesting tinnitus as a unified percept of interacting separable subnetworks, the OFC has been suggested to be one of the cortical areas responsible for the emotional component of tinnitus (De Ridder et al., 2014). In addition, recent studies demonstrated that tinnitus-related distress is related to alpha oscillation over the network consisting of the amygdala-anterior cingulate cortex-insula-PHC (Vanneste et al., 2010; Golm et al., 2013). Moreover, another study demonstrated a correlation between tinnitus-related distress and sympathetic activation, mediated in part via the right anterior insula (van der Loo et al., 2011).

These previous studies were consistent with the present findings showing positive correlations between cortical power changes in the right insula/OFC and the percentage improvements in NRS distress 6 months after the initial TRT session. That is, greater normalization of OFC-mediated emotional components and right insula-mediated sympathetic activation by TRT showed an association with greater improvement in tinnitus-related distress. In addition, our preliminary study revealed a positive correlation between the pre-TRT activity of the insula and the percentage improvement in THI score, reflecting the improvement of psychological distress due to tinnitus, 3 months after the initial TRT session. Taken together with the results of our preliminary study, the findings presented here suggest that TRT may be more successful in subjects with severe distress due to abnormal autonomic nervous system activation.

### Strengths and Limitations of the Current Study

To our knowledge, this is the first study to evaluate cortical power changes after TRT and to correlate these changes with improvements in subjective symptoms of tinnitus. Although this study not only replicated previous findings but also elucidated key changes responsible for the improvement of tinnitus and related distress, there are several potential limitations that warrant follow-up investigation.

First, we processed qEEG data by resampling to 128 Hz and band-pass filtering with the standard EEG wave range (2–44 Hz) in this study. A previous study, however, showed higher frequency (40–80 Hz) activities in the gamma range over auditory cortices in patients with intractable tinnitus (Ashton et al., 2007). The precise interpretation of the changes in gamma oscillation, which have been suggested to be neural correlates of perception and consciousness (Weisz et al., 2007), was not possible in the present study. Therefore, further studies using higher frequency band recording are required. Second, although we reported improvements in tinnitus-related symptoms 6 months after modified TRT, we cannot definitively conclude that these improvements were solely due to TRT. As tinnitus resolves spontaneously without any treatment during follow-up in some patients (Dobie and Snow, 2004) and as it is recommended for the TRT program to be continued for 18 months (Grewal et al., 2014), further studies comparing a TRT group with a “waiting list” control group with a longer follow-up duration are warranted. Third, tinnitus patients with relatively high levels of distress (mean THI score of 57.8) were enrolled in the present study. To explore the applicability of the present results to patients with different levels of distress, further studies in subjects with no or mild distress are required. Fourth, we enrolled tinnitus patients with normal hearing or only mild hearing loss and did not take combined hyperacusis into consideration. It has been reported that tinnitus patients show different cortical activity patterns according to the degree of hearing loss or combined hyperacusis (Vanneste and De Ridder, 2016). Therefore, further studies to explore the cortical power changes that occur after TRT in groups with different degrees of hearing loss and to evaluate the role of hyperacusis are required. Fifth, a recent study has suggested that there were no differences with regard to tinnitus improvement scores among TRT, partial TRT (counseling with placebo sound generator), and standard of care groups (Tinnitus Retraining Therapy Trial Research Group et al., 2019). Further research is warranted to reveal whether or not the effect of TRT is in a part of natural habituation. Sixth, our study subjects were heterogeneous with regard to tinnitus laterality (16 bilateral tinnitus, 15 right tinnitus, and 6 left tinnitus). In this regard, the findings in the current study are inconclusive as unilateral- and bilateral tinnitus subjects show different cortical oscillatory patterns according to previous literature (Vanneste et al., 2011). Future studies exploring different effects of TRT both in uni- and bilateral tinnitus groups should be performed to further explore the effect of tinnitus laterality.

## Conclusion

Taken together, the results presented here indicated that TRT improves tinnitus by reducing the auditory cortical power and tinnitus-related distress by reducing the power of the top-down autonomic response modulator or peripheral physiological responses to emotional experiences. That is, TRT induces habituation via modulation of the functional connections between the auditory system and the limbic and autonomic nervous systems. The present study confers an additional basis for understanding the neurophysiological model and the newly suggested integrative model of tinnitus by De Ridder et al. (2014) in the context of the long-term efficacy of TRT. In addition, the present study suggested a mechanism by which TRT quiets tinnitus and relieves tinnitus-related distress in the context of psychological modulation of cortical power.

## Data Availability Statement

The datasets generated for this study are available on request to the corresponding author.

## Ethics Statement

The study was approved by the Institutional Review Board of the Clinical Research Institute at Seoul National University Bundang Hospital and was conducted in accordance with the Declaration of Helsinki (IRB-B-1708-412-128). The requirement for patient consent was waived.

## Author Contributions

J-JS led the analysis and interpretation of the results, and drafted the first manuscript. S-YL, JR, and YS conceived the investigation, revised the manuscript for important intellectual content. All authors contributed to all aspects of the investigation, including methodological design, data collection and analysis, interpretation of the results, and revision of the manuscript for important intellectual content, and approved the final version of the manuscript and agreed to be accountable for all aspects of the work.

## Conflict of Interest

The authors declare that the research was conducted in the absence of any commercial or financial relationships that could be construed as a potential conflict of interest.

## Footnotes

1 http://www.uzh.ch/keyinst/NewLORETA/Software/Software.htm
